# Material Engagement Shaping Participation of Children on the Autism Spectrum: Embodiment and Subjectivity in Small-Group Learning

**DOI:** 10.5964/ejop.13307

**Published:** 2024-05-29

**Authors:** Juliene Madureira Ferreira, Luciana Soares Muniz

**Affiliations:** 1 Faculty of Education and Culture, Tampere University, Tampere, Finland; 2 Teacher Education and Training School, University of Uberlândia, Uberlândia, Brazil; Concordia University, Montreal, Canada

**Keywords:** materiality, embodiment, participatory sense-making, autism research, basic education, participation

## Abstract

This study investigated the material engagement and their affordances for participation of children on the autism spectrum (AS) in small-group learning. Framed by a methodology called Idea Diary that fosters social interactions in classroom environments, our focus was on understanding how and when the construction and manipulation of the diary supported children’s participation and knowledge construction in small groups. This investigation was guided by the intersection of the theory of subjectivity developed by Fernando González Rey and enactive accounts of cognition. This framework provided the view of the singularity in the communicative process of children on the AS and the necessary support for examining the mechanisms of engagement that led to children’s participation. We present two case studies of 9–10-year-old boys. Data consists of the diaries produced and used by children and video recordings of children’s interactions during small-group discussions. Our analytical approach included a qualitative semiotic analysis of the materials and a micro-analysis of the social interactions. The results showed, first, that children on the AS continuously engaged in the construction of the diary, expressing elements of their subjectivity—experiences, ideas and the system through which they interact with the world. Repetition framed children’s productions and signalled engagement. Second, material engagement enabled participatory sense-making, which in this study appeared in creating new communicative resources between the child on the AS and their peers and in adapting the narratives, approximating children’s perspectives in conversations. Although contextualised within a specific pedagogical practice, the study contributes to advancing our understanding of the role of material engagement in social participation in learning situations involving children on the AS, particularly relevant in educational psychology and education.

In the past decade, autism research has significantly advanced to explore and better understand sociality among people on the autism spectrum (hereafter AS^1^1The language used to refer to people who experience living on the autism spectrum can vary according to norms in different socio-cultural contexts and personal preferences. In this study, we follow the “person first” language recommended by the Autism Spectrum Finland (Suomen Autismikirjon Yhdistys - ASY) and the Association of Friends of Autism (Associação dos Amigos dos Autistas - AMA), although recognising that some adults in these countries prefer “identity first” language (autistic person).) (Ochs & Solomon, 2010; Sterponi, 2017; Sterponi & Shankey, 2014). Moving away from comparing communicative performances against ‘standard’ neurotypical interactions, the contemporary research in the field has aimed to characterise the unique linguistic resources people on the AS bring to interactions and see how they are deployed in service of interactional aims (Bottema-Beutel, 2017; Sterponi et al., 2015). By employing methods that focus on the structure of communication dynamics such as Conversation Analysis (Sidnell & Stivers, 2012) and supported by embodied theories that explain the mechanisms underlining social interactions by the enactments within the interaction (De Jaegher, 2021; Fantasia et al., 2014), studies have given visibility to the communication competencies of people on the AS, unravelling the different engagement patterns that may be dismissed as pathologies (Ferreira & Bottema-Beutel, 2023; Yu & Sterponi, 2023).

Understanding the sociality of people on the AS is the most respectable and efficient way to guarantee their participation in social contexts (De Jaegher, 2021). However, communication (verbal and non-verbal) is only one element constituting participation in social interactions. Our bodies are grounded in a specific time, space, and materiality that shapes our cognition and affords our actions in the world (Gallagher, 2004, 2005). Furthermore, the mere way we engage with objects (things around us) can change our experience in action and the meaning we create of such experiences (Malafouris, 2018a). Therefore, participation is a complex process of enacting and co-constructing meaning, fundamentally dependent on the material world around us. Despite the current advances, research investigating how people on the AS use and co-construct material culture within social interactions is scarce.

Recognising the role that material culture plays in shaping human development and its potential to foster new opportunities for interaction across multiple timescales and social contexts, the present study explores how material engagements can shape the participation of children on the AS in small group learning situations. This investigation is framed by a specific pedagogical methodology designed to support small-group learning called *Idea Diary* (Muniz, 2015). This method uses a material resource—a diary—as a tool to support children in *experiencing, recording, and sharing* their ideas, interests, and learning processes through multiple languages of expression. Our theoretical standpoint intersects a cultural-historical perspective on subjectivity that emphasises its role in the schooling process (González-Rey & Mitjáns-Martínez, 2017) and an enactive account of cognition (De Jaegher & Di Paolo, 2007), which guides our investigation and understanding of interactive and material engagement processes that will set participation and learning in small groups. Together, these theoretical frameworks support the exploration of how elaborating on the diary allowed children to express elements of their subjectivity, participate, and learn in small-group interactions.

## Social Participation and Material Engagement in Autism

The autism spectrum is a lifelong developmental condition related to persistent difficulties initiating and sustaining communication and social interaction and a range of restricted, repetitive, and inflexible behaviours, interests or activities (DSM-5: American Psychiatric Association, 2013). Children on the AS experience the social and sensory world around them differently than their neurotypical classmates (De Jaegher, 2021), encountering multiple barriers to participation in learning situations, especially when expected social acts to be like those in neurotypical children. Participation is a complex phenomenon, often identified by the physical presence, social interactions with others and the environment, and active engagement in learning activities (Ferreira, 2017). It is key to ensuring inclusive schooling for all children, including those with significantly different developmental paths (Black-Hawkins et al., 2007). Many educational programs target social skills development in children on the AS to enhance their participation in school activities and, thus, in learning (e.g., Anderson & Meints, 2016; Mpella et al., 2019). Nevertheless, although such programmes suggest a positive effect on social responsiveness (Dubreucq et al., 2022), they rarely focus on understanding children’s subjectivity and how they express themselves in the school context. Rather, typical behaviours determine social skills, lacking a focus on actual social participation (Płatos & Wojaczek, 2018).

Furthermore, current research evidences significant architectural and brain-functional differences between people on the AS and neurotypical people (O’Reilly et al., 2017), supporting the argument that their engagement with the world is affected by their condition (Wright et al., 2022). This is consequential in understanding affordances for material engagement and its role in shaping cognition. For example, studies have found a broad attentional spotlight and enhanced perceptual function in individuals on the AS compared to typically developing adolescents (Smith & Milne, 2009). This could explain the feelings of sensory overload often described by people on the AS and the expression of adverse behaviours when having to deal with overstimulated environments. Studies have also investigated visual attention patterns and identified a preference for non-social over social stimuli in preschoolers on the AS (Sasson & Touchstone, 2014) and an ability to understand complex patterns (Baron-Cohen, 2009), which can both impact various situations relevant to children’s development, especially at school (e.g., in collaborative play, preference for specific topics or learning settings). Following these insights, individuals with AS may engage in material culture uniquely. Differences in material engagement can be as significant as differences in social engagement. However, what we see across most research in psychology and education is an overwhelming confirmation of the limitations of people on the spectrum in engaging with the world—socially and materially. The efforts to investigate the processes circumscribing these diverse and distinct engagement experiences are scarce, especially in the school environment where, although the diversity of children’s developmental paths co-exists, material practices are standardised.

The reason behind this situation is twofold. First, traditional cognitivist theories explain the ability to understand others and participate in social interactions through the ability to infer intentions and predict others’ actions, creating a representational action understanding system (Cannon & Woodward, 2012). This ability is assumed to be limited among children on the AS, hindering their participation in social encounters (Fantasia et al., 2014). Mainstream theories also dismiss the implications of even less radical notions of material engagement (actions guided by our perception of objects) in cognition, considering the relation mind-body-environment a trivial one (Malafouris, 2018b). Second, most research in psychology and education examining interactions and learning among people on the AS is conducted in artificial settings, usually focusing on methods to identify characteristics and compare social behaviours, learning difficulties, and cognitive development in autism spectrum conditions. This approach often reproduces, even unintentionally, an emphasis on the limitations of children on the AS (Fantasia et al., 2014). Less research explores the strengths and capabilities of children on the AS (e.g., Dindar et al., 2016) and how to apply this knowledge in educational settings (White et al., 2023). Therefore, by assuming that children on the AS will, from the get-go, ‘fail’ in social cognition, research and educational programs are discouraged from developing methods and practices addressing this process. Consequently, there are fewer opportunities for people on the AS to express themselves and be understood by their ways of communicating their lived experiences in and with the world—to be validated in their subjectivity. To remedy this situation, the present study focused on understanding how children on the AS engage with materials and use them to communicate elements of their subjectivity, participating in small-group interactions at school.

## Subjectivity and its Role in Learning Processes

While learning is a social act, it also depends on individual subjectivity (Oliveira & González Rey, 2018). We adopt the concept of subjectivity developed by González Rey (2016), which sees it as a complex way of understanding human psychological functioning within the concrete/material conditions of culture. Subjectivity is the configuration of symbolic-emotional units comprising the feelings and senses we make of our experiences in the world—subjective senses (González Rey, 2003). Thus, it is inseparable from human actions and is constructed (like a kaleidoscope) in the continuum of our social interactions in different spaces and social spheres. These subjective senses are created in the moment of action—engagement, in the material world and the social systems (e.g., family and school) embedded in it. Subjectivity is then understood as the assembly of these subjective senses forming a psychological system that determines the individual’s future engagements in the world in a dynamic and continuous process. This notion of subjectivity, different from other approaches, highlights the emotional and generating character of individuals concerning what they experience. Humans are not passive or merely assimilating what is happening to them. Emotion becomes sensitive to symbolic records, allowing the individual to generate an understanding of the world that enables them to transcend the immediate context of actions through their subjective productions (González Rey & Mitjáns-Martínez, 2017).

Subjectivity will then guide and participate in children’s learning experiences. The child arrives at school with specific subjective configurations from previous experiences in multiple social contexts. For example, when children learn about the solar system, they bring subjective senses (symbolic and emotional units) to the classroom about it constructed in their previous encounters with this topic (e.g., from YouTube videos or from observing the sky with their parents). These historical configurations of subjective senses will be part of the learning experience of knowledge construction about the solar system in the school context. In describing the solar system in a learning activity, subjective senses related to, for example, the fascination for planets, fears of the notions of the infinite, or admiration for the beauty of space can emerge. Thus, each learning process is individual, and there are no patterns of relationships that will define the quality of learning; it is how the learner configures subjective senses of the learning situation that defines the learning action (González Rey & Mitjáns Martínez, 2017; Muniz & Mitjáns Martínez, 2019). This notion of subjectivity considers the permanent interrelationship between the individual and the social, as well as between the historical and the present moment. Thus, it encourages the investigation of the elements of children’s subjectivity configuring learning situations, emphasising the expressions of emotionality permeating the learning process (Medeiros & Goulart, 2022).

## Embodiment and Materiality Shaping Participation and Learning in the School Context

Embodied cognition (EC) theories challenge (to different degrees) the idea that our mind is in our brain and clearly separated from our body and the environment. It suggests that cognitive processes, such as memory, perception, action planning, and understanding others’ actions (among others), involve sensory-motor systems, bodily movements, and actions with the world. Therefore, our experiences and perceptions are partly enabled by our bodies and their specific physiological structures (functioning) (Uithol & Gallese, 2015) and shaped by the material affordances available in the environment. Embodied approaches (including Embodied, Enactive, Extended, Embedded, and current developments in Ecological-enactive frameworks) explain the mechanisms underlining social interactions by the enactments within the interaction man-world instead of solely by mentalistic and representational theories (e.g., Theory of Mind) that confine the ability to co-construct meaning with others and experience shared intentionality to sophisticated mind representation of other’s states of mind (De Jaegher, 2021; Fantasia et al., 2014).

Under this theoretical framework, specifically grounded in enactive approaches to cognition, we reflect on participation and group learning processes through the lenses of *participatory sense-making* (De Jaegher & Di Paolo, 2007). In brief, sense-making is a unique capacity of human beings through which we signify or establish a perspective on the world. It is a process led and regulated by our need to preserve our autonomy and self-organisation as living systems (Di Paolo et al., 2018). Participatory sense-making is the interaction in which establishing perspectives of the world is constructed with others. It is both the definition and the operationalisation of the role of the interaction in the cognitive process and what gives humans an agentic nature (De Jaegher & Di Paolo, 2007). The relationship between the individual and the world is enacted (brought forth) through actual engagement (De Jaegher & Di Paolo, 2007; Kyselo, 2016). Through this perspective, understanding how participation in small-group interactions is constructed and leads to learning situations demands that we look towards (1) what is being created within (“in between of”) the interactive processes rather than the individuals’ behaviours during the interaction, and (2) how the material environment affords such engagement and how this engagement builds on meaning-making. The things we make and how we use them not only reflect our beliefs about the world but also affect how we understand the world (Malafouris, 2018a, 2018b). The constitutive role of objects in cognitive development is already identified in early childhood. For example, recent developmental psychology studies have shown the fundamental role of the canonical use of objects in concept development and in intention understanding in early childhood (Alessandroni, 2021; Vietri et al., 2022). Therefore, we understand that materiality shapes our engagement with the world, affording (or not) participatory sense-making.

Furthermore, enactive approaches account for social cognition as processes sustained by embodied coordination, which do not necessarily require cognitively complicated skills (e.g., mind reading). Instead, sense-making is constructed through the engagement—enactment—within the interaction (De Jaegher & Di Paolo, 2007). This premise is particularly important in autism research as it enables the starting point of an analysis of social interactions among people on the AS to be the unique ways of that person to relate instead of their possible limitation in social cognition. Thus, the enactive framework is a potent approach for investigating social interaction as it brings insights into the dynamic between peers that establish coordinated efforts to learn together (De Jaegher, 2013; Fantasia et al., 2014). In this study, enactive theories complement the theory of subjectivity by providing further insight into the mechanism of cognitive processes, such as how material engagement through action builds a specific cognitive repertoire.

## The Present Study

In the present study^2^2This study is part of a larger research project exploring the participation and agency of students in special education, targeting the development of embodied and inclusive pedagogical practices. For more information on the project, access: https://research.tuni.fi/ecepp/research/child-development-and-diverse-needs-in-early-years/embodiment-intersubjectivity-and-the-construction-of-childrens-agency-in-learning/, we examined the material engagement of children on the AS in small-group learning in two different socio-cultural and educational contexts—in Brazil, within a regular classroom and in Finland, within a special classroom. The study was framed by implementing a specific pedagogical method, *Idea Diary*^3^3*Idea Diary* in italic referes to the methodology. Idea Diary in normal text refers to the notebook–the diary, constructed by children during the learning process promoted within the methodology., during the 2021–22 academic year in both countries. The *Idea Diary* is grounded on the Theory of Subjectivity proposed by Fernando González Rey (González Rey & Mitjáns-Martínez, 2017). It is designed specifically to facilitate children on the AS to express themselves, their ideas, interests and how they make sense of the world—a pedagogical approach constructed for studying subjectivity. Within this context, we particularly explored the material affordances for participation, aiming to understand if and how materiality supports children’s participation and how children on the AS construct, manipulate, and use the diary to communicate their ideas, experiences, and interests with other children. To guide the study, we asked: How do children on the AS construct and use the diary to express their ideas, interests, and experiences? How do children on the AS position themselves and invite others to participate in small-group discussions?

### The Methodology of the Idea Diary

The Idea Diary, developed by Muniz (2015, 2020, 2022), supports creative literacy learning in classroom interactions. It is a pedagogical tool that provides a clear structure for children to reflect on their learning interests, experiences, and knowledge, encouraging them to associate learning in different social contexts outside the school. It also supports teachers in systematically accessing and understanding elements of children’s subjectivity emerging from students’ reflections (Muniz & Mitjáns Martínez, 2019).

The *Idea* aligns with the overall understanding that creativity is the product of children’s actions in the face of what is experienced and an expression of human subjectivity (Mitjáns Martínez, 2012). Creativity is understood as a process of human subjectivity that can be expressed in learning through the person's actions concerning what they learn. It is a singular expression, a subjective production of the person in the context of action. Thus, there is no linear relationship between how pedagogical work is carried out and the expression of creativity. However, creativity can be enhanced by school actions that value students' authorial and protagonist expression and by personalising teaching and learning (Muniz & Mitjáns Martínez, 2019). Children express creativity when they confront their reality, raising doubts and questions about what they learn; when they understand the impacts of what they have learned on a person’s life; when they generate new ideas beyond what is experienced, and fourth, when incorporating a playful relationship with learning—a spontaneous and free commitment to learning (Muniz & Mitjáns Martínez, 2019).

The *Idea Diary* connects three fundamental actions: Experiencing, recording and sharing. It brings forth children’s sensorial learning (e.g., through touching and feeling) and enactments in the world, and by providing a material space for children to note their experiences, it creates the necessary support for reflection during the learning process. The method entails using a notebook—the diary, as a “place” for children to collect their ideas about what they are learning or experiencing that is significant to them. Children record ideas they would like to develop further or topics they became interested in as they experienced something new. They can express themselves and their worldviews through writing, collages, drawings, attaching photographs, pictures, or any other material they believe is important for expressing the idea and composing the action of sharing. Supported by the diary, children share their memories of the experience in the group, bridging their inner world to the worlds of others.

The diary also becomes material for teachers to investigate children’s learning and expressive processes and represents the multiple possibilities of implementing the principles for achieving learning as a subjective process (for a complete view of pedagogical applications of the Idea Diary see Muniz, 2022). For this method, ideas are not external to people, but processes loaded with emotionality and express the person's generating character in the face of what they experience. In this way, all children may feel that they belong to the group, recognising the life story of each learner participating in learning. The teaching and learning process is carefully personalised and built with the teachers' and students’ active participation in aligning the learning objectives and pedagogical actions.

**Figure 1:**
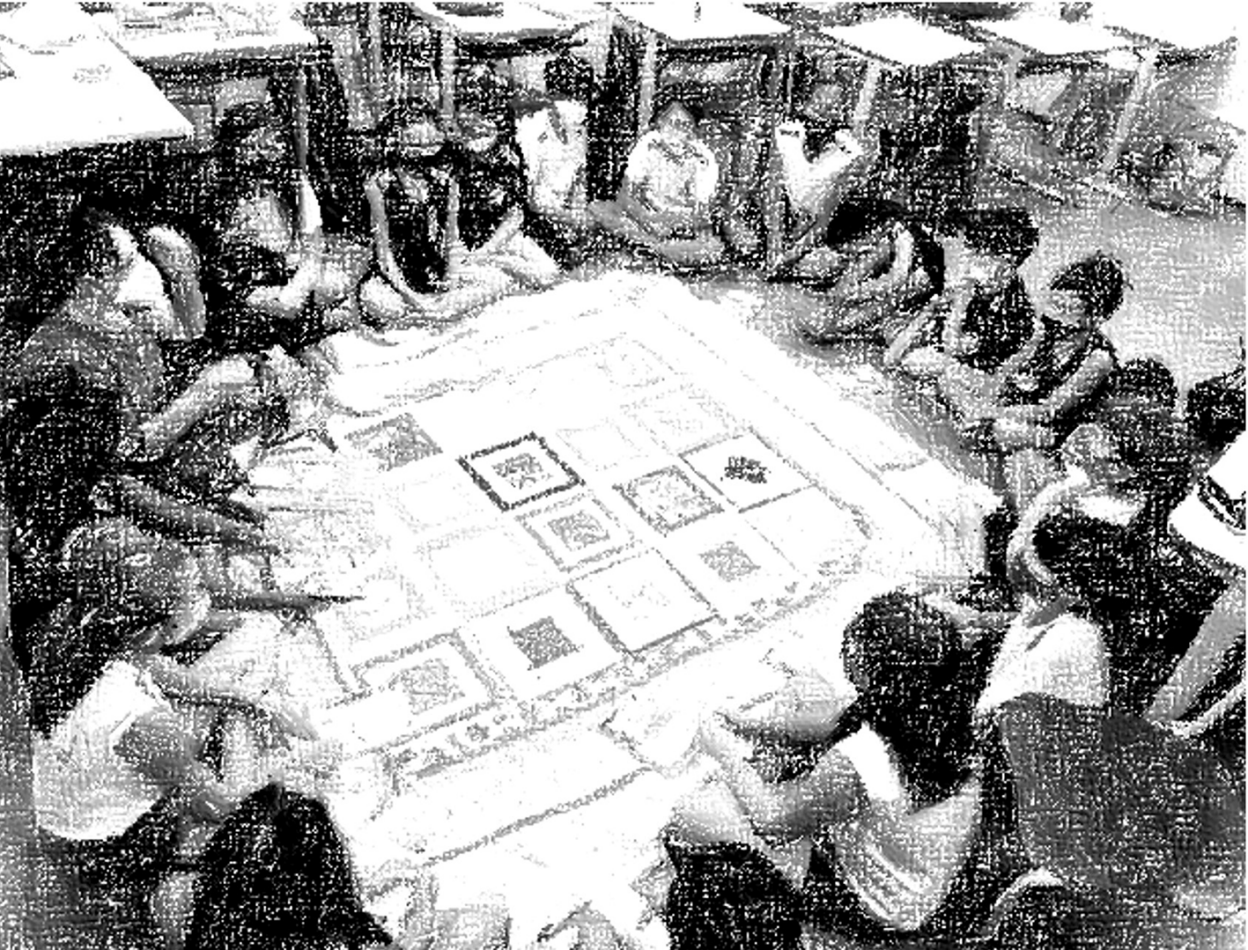
Illustration of a Sharing Circle

The practice begins with the teacher introducing the idea of the diary, usually using storytelling to engage children’s attention. The story is about a pirate sailing across different lands, collecting interesting experiences. He collects and saves these experiences, represented by words kept in a chest. Once the diary is introduced in the class, children are instructed to take the diary periodically (e.g., once or twice a week, depending on the group), and the group gathers in morning circles to discuss each child’s experiences and ideas. The teacher invites the children to share what they have produced in their diaries (see Figure 1). The discussions in the morning circle are transformed into learning topics, allowing the teacher to use this knowledge in preparing the school curriculum and potentialising creative learning in the classroom^4^4Detailed methodology of the Idea Diary is available on the website http://www.lucianamuniz.com.br/metodologia/.

Teachers organise and conduct sharing circles. The dynamic in the *Idea Diary* privileges space-time for inquiries, exchange of ideas, valuing children’s experiences and knowledge in a dialogical approach (Freire, 2011; González Rey & Mitjáns Martínez, 2017), and keeping children’s epistemic authority of the knowledge (Heritage, 2012). The discussions supported by the materiality of the diary (i.e., pictures, drawings, writings, or collages) allow children to elaborate on their learning experiences and interests, knowing they will be shared, which we believe can progressively structure the interaction toward constructing participation.

## Method and Materials

This qualitative case study (Yin, 2018) brings forth two expressions of the material-cultural affordances for participation and learning among children on the AS available during the implementation of the *Idea Diary*. The first case is of a Finnish 10-year-old boy, Alan, who regularly attended school under full-time special education support in a special classroom. Alan’s school is a regular public school in the Pirkanmaa area, South Finland, that receives around 770 students from pre-school (6 years old) to 9th grade (15 years old) yearly. It has a team of four special education teachers and four professionals responsible for students' well-being and development, including a psychologist and a social worker. This is one of the few schools in the area that still maintain special education classrooms. In Finland, special classrooms are the less common resource of special support. Special support is the third and final level of the three-tier educational support service offered to students in the Finnish Inclusive Education System (see EDUFI – Finnish National Board of Education, 2016; Ferreira et al., 2022). It often involves material adjustments, part-time small-group or individualised lessons with special education teachers, or a flexible curriculum aiming to secure children’s learning and academic achievement in the schooling process (OSF – Official Statistics of Finland, 2020).

The second case is of a 9-year-old boy, Henrique, from Brazil. Henrique attends school part-time (four and a half hours) in a regular class at a public school in Uberlândia, Minas Gerais, Brazil, and receives two hours per week of special education support in the afternoon shift. The school receives 800 students from early childhood education (4 years old) to 9th grade (15 years old). The school employs a team of four special education teachers, four educational psychologists and one social worker, all responsible for the development and wellbeing of the students. In Brazil, specialised educational support [Atendimento educational especializado] consists of individual or small group classes with a special education teacher, organised mainly in the after-school period. During school hours, the child participates in regular classes, monitored by a class aid under the supervision of the special education teacher. Curricular flexibility can be applied as a final resource and only with the family's consent (for more information, see Dias et al., 2019; Lustosa, 2018). The description of the developmental specificities and pedagogical notes are available in Table 1.

**Table 1:** Configuration of the Case Studies

Case	Age	Developmental specificities according to school records	Summary of pedagogical notes provided by the teacher
Case 1. Alan	10	Was diagnosed with Autism Spectrum Disorder at an early age. Presents repetitive behaviours, considerable difficulties in establishing social interactions and sensorial sensitivity.	In the classroom, he engages mainly in individual activities and often stays in his space in the classroom. He verbalises his wishes when he wants and answers direct questions but does not initiate social exchange. He remembers many things that he has read and repeats verbally the contents he has learned. He expresses repetitive body movements for self-regulation and cacophonies in different moments of the classroom routine. He is learning and interested in school. Interested in trains and calendars.
Case 2. Henrique	9	Was diagnosed with Autism Spectrum Disorder at an early age. He is a non-verbal child with difficulties expressing his emotions, showing hypersensitivity to changes and reacting negatively (signs of stress) to overstimulating environments and/or when others do not understand his needs.	In the classroom, he has difficulties engaging in school activities and often walks around the class between other students’ chairs. He is learning to interact with others through gestures and movements engaging other’s bodies. He likes bright objects, music and children's videos but has significant language limitations, challenging his full participation in the classroom.

*Note*. Information on each case was provided by the class teacher based on school records. The researchers did not administrate any standardised testing for this study.

The purpose of bringing cases from these two different socio-cultural contexts is to give visibility to different appropriations of the diary as a material-cultural instrument for participation rather than comparing practices and cultural specificities, which will be addressed in another opportunity.

### Data Collection

In Finland, the data was collected by the first author during 22 consecutive weeks of the academic year 2021. The Idea Diary was implemented by the class teacher, assisted by a class aid, who was trained to implement the *Idea Diary* method before the beginning of the study. During the first two weeks, the first author immersed herself in the research site, allowing familiarisation with the students as recommended in previous studies (Heath et al., 2010). The *Idea Diary* practice continued for 20 weeks with at least one morning circle and one collective activity per week, totalising 11 hours of material, from which 240 min were specifically about the sharing circles. In Brazil, data was collected by the second author, assisted by two research assistants. In this context, the second author was also the class teacher implementing the *Idea Diary*, thus with complete familiarity with the method and the functioning of the class. The *Idea Diary* was carried out biweekly during the entire academic year 2022, also resulting in 20 weeks of practice and generating 10 hours of video, of which 190 min were specifically on the sharing circle.

In both cases, children were instructed to take the diary home for two days before each morning circle and freely produce entries in the diary. Children’s parents were instructed to support children, if needed, to engage with the diary at home, for example, following children’s instructions and helping get materials. Children could build on the entries using diverse materials and methods, such as drawing, written texts, collages, photographs, or even videos. Nevertheless, they could not interfere with the production of the diary (i.e., drawing or writing on behalf of the child). All morning circles were video recorded following recommendations on using mobile cameras operated by the researchers (Ukkonen-Mikkola & Ferreira, 2019). Parallelly, both researchers kept ethnographic field notes (Emerson et al., 2011) entailing details about the spaces, timings, daily routines, and reports of feelings, ideas, impressions, and hypotheses for explaining the observed dynamics that could support understanding children’s meaning-making process. After each morning circle, researchers photographed children’s idea diaries and catalogued all entries, generating a pictorial dataset comprising 47 and 20 entries in Alan and Henrique’s diaries, respectively.

### Analysis

To understand how materiality supports children’s participation and subjective expressions, we developed an analytical approach that allowed us to focus on analysing the materials produced by the children and how children used these materials in social interactions. We combined semiotic analysis of children’s material production (Deguara, 2019) and microanalysis of video recordings (Heath, 2004; Heath et al., 2010) of interactional meaningful events. This approach differed from the constructive-interpretative method usually applied in subjectivity studies (González Rey & Mitjáns-Martínez, 2017) to allow for a closer look at the material affordances of the Idea Diary and a more fitting approach to studying the unfolding of non-verbal behaviours. Also, while constructive-interpretative analysis is better applied in parallel to data construction, in this study, we approached the analysis after completing the Idea Diary implementation in both countries.

**Figure 2:**
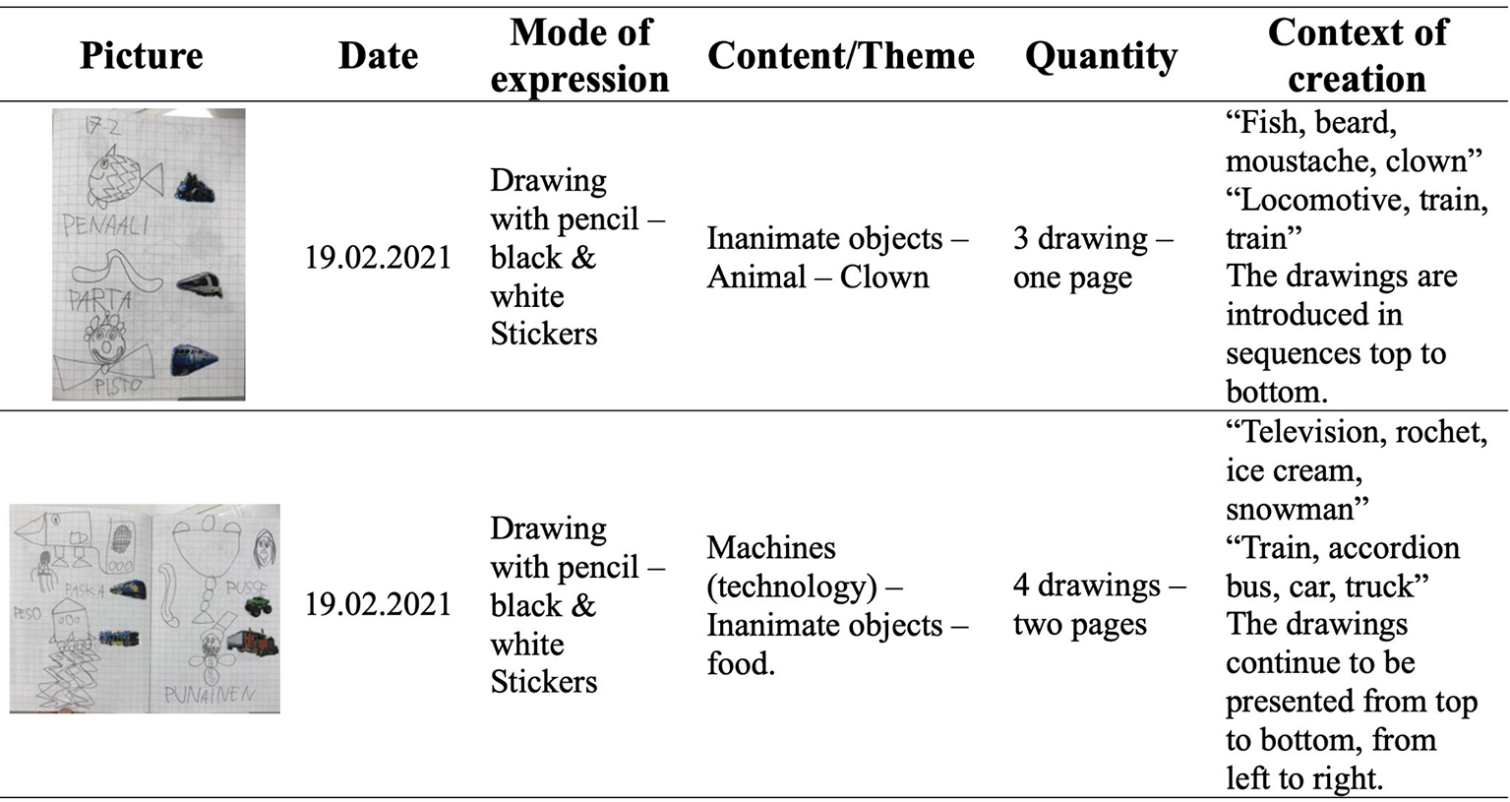
An Example of how the Grid From the Semiotic Analysis is Constructed

The investigation started with the semiotic analysis of children’s entries in the idea diaries, which entailed examining the type and quantity of the modes of expression used by the children (i.e., the form of the drawing, collage, or written expression) and the variety and quantity of the themes (i.e., content) expressed by them (Deguara, 2019). Both perspectives are then juxtaposed, creating a grid that reflects children's preferred semiotic (modes) and configuration (themes) styles (see Figure 2). This analysis allows for observing how children choose to express themselves and provides an overview of children’s interests, experiences, and ideas.

After mapping the semiotics, we focused on identifying the patterns and the transitions in the modes of expression and types of themes. The first provided an overview of children’s general interests, affinities, knowledge, or experiences. The latter indicated the potential emergence of divergent ideas, sharing significant events, introducing new expressive repertoire, or prompting specific actions. Thus, it should also be further explored through the microanalysis of children’s interactions in small-group discussions.

The subsequent step was the microanalysis of the videos (Heath, 2004). Following a preliminary scanning of the video materials, we catalogued 24 candidate fragments as meaningful events for further analysis, 11 from Henrique and 13 from Alan. These meaningful events consisted of moments when Henrique and Alan, within interactions with their respective peers, participated in the dynamic of the morning circle (e.g., creating resources for communication, jointly changing the representation of the ideas, experiences, or information in the diary, or initiating a specific group action). The microanalysis was supported by ELAN software ​​(Brugman & Russel, 2004, Version 6.5, 2023), through which we carefully coded each child’s behaviours, including gaze, body posture, movements, and actions, creating a timestamp of unfolding events during small-group interactions. We particularly looked for moments of embodied coordination generating specific meanings in the moment of the interaction.

### Ethical Considerations

This study received ethical approval from the Tampere Municipality’s Institutional Review Board before the commencement of the study (protocol under the name of the first author) and the approval of the Ethical Committee of the Federal University of Uberlândia (protocol CAAE: 91490518.7.0000.5152*)*.

The study followed all recommendations for researching children from both countries and in consideration to what is internationally expected, according to Bertram et al. (2016) and the Finnish National Board on Research Integrity. Anonymity in this report is ensured by the use of pseudonames and imaging filters of children’s faces. Parents’ consent was acquired before the study, and children’s consent was gathered throughout the process. The researchers were sensitive to signs of children’s distress or unwillingness to participate according to ethical recommendations when using video recordings (Ukkonen-Mikkola & Ferreira, 2019). Two children from Finland whose parents did not consent were not recorded. Respecting the parents’ decision, we scheduled the video recording of small-group discussions to coincide with these students' extra-class activities. Children not engaged in the study were, however, participating in the other activities of the *Idea Diary* and were included in all learning opportunities that derived from the shared discussions. During small group discussions, researchers showed the children how the camera worked, briefed them about the duration of the videos, and allowed them to manipulate the instruments and ask questions. Parents did not consent to the reuse of data or the publication of images identifying their children. The data management plan and metadata of all video recordings are available upon request from the first author.

## Results

Meaningful events appeared throughout all small-group interactions recorded during the *Idea Diary’s* implementation. Children engaged physically or verbally in all morning circles, making their presence noticed and valuable contributions to the group’s dynamic. We summarise our results in two important observations that answer the study’s research questions: (1) The patterns and transformations in children’s expression and communication and (2) The material engagement supporting self-perception, shaping participation, and enabling participatory sense-making. To support the presentation of results, we chose examples that illustrate how children on the AS construct and use the diary to express their ideas, interests, and experiences and how they position themselves and invite others to participate in small-group discussions.

### The Patterns and Transformations in Children’s Expression and Communication

**Figure 3:**
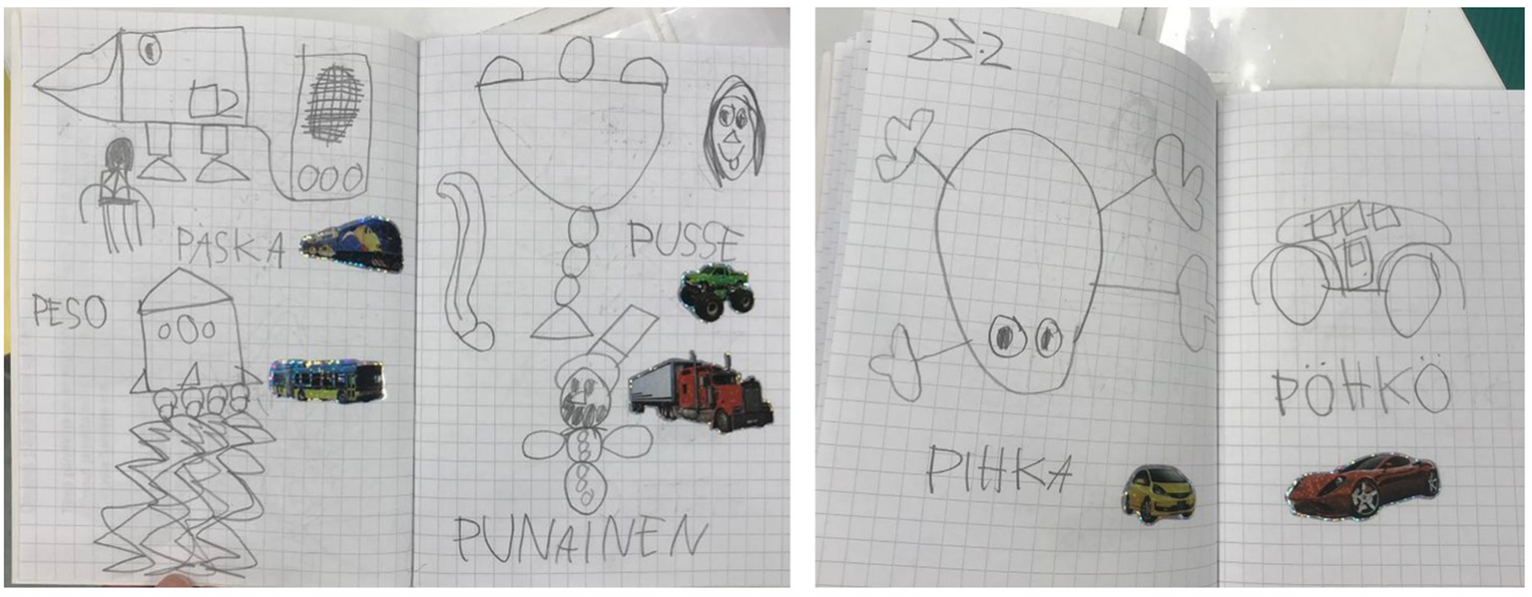
Engagement Through Repetition

In Alan’s diary, the use of words starting with the letter P and the same mode of expression—through black and white drawings, marked the repetitive pattern in children’s expression of their experiences and ideas (Figure 3). Although the drawings varied significantly, bringing together representations of objects, places, characters, and food, the presentation of the illustrations kept a certain order and aesthetics: a black and white free-hand drawing, a word tag (not necessarily representative of the drawing) and a sticker of the child’s favourite theme—vehicles and trains. Much like the story introducing the *Idea Diary* in the classroom, Alan’s diary reassembled a collection. Instead of letters, he collected drawings, words, and stickers that were kept together by the constraints of the notebook, showing his commitment to the activity consisting of *experiencing, recording,* and *sharing* with the group.

When the teacher inquires about the student’s reasoning behind the drawings and the use of the letter P, Alan reveals that he makes notes of things that come to his mind: “They (drawings) are inside my head”, and that the letter P is a reference to one passage of the pirate’s story used to introduce the *Idea Diary*: “I write a different word below each picture (...) because you have said (referring to the story) *‘what will happen if all names start with the letter P?’”*. In this meaningful event, we see that engaging with the diary allows Alan to (re)live the experience of the story, and every time he makes an entry in the diary, he brings forth a way of structuring his ideas and the elements of subjectivity that he wants to share with others guided by that experience.

**Figure 4:**
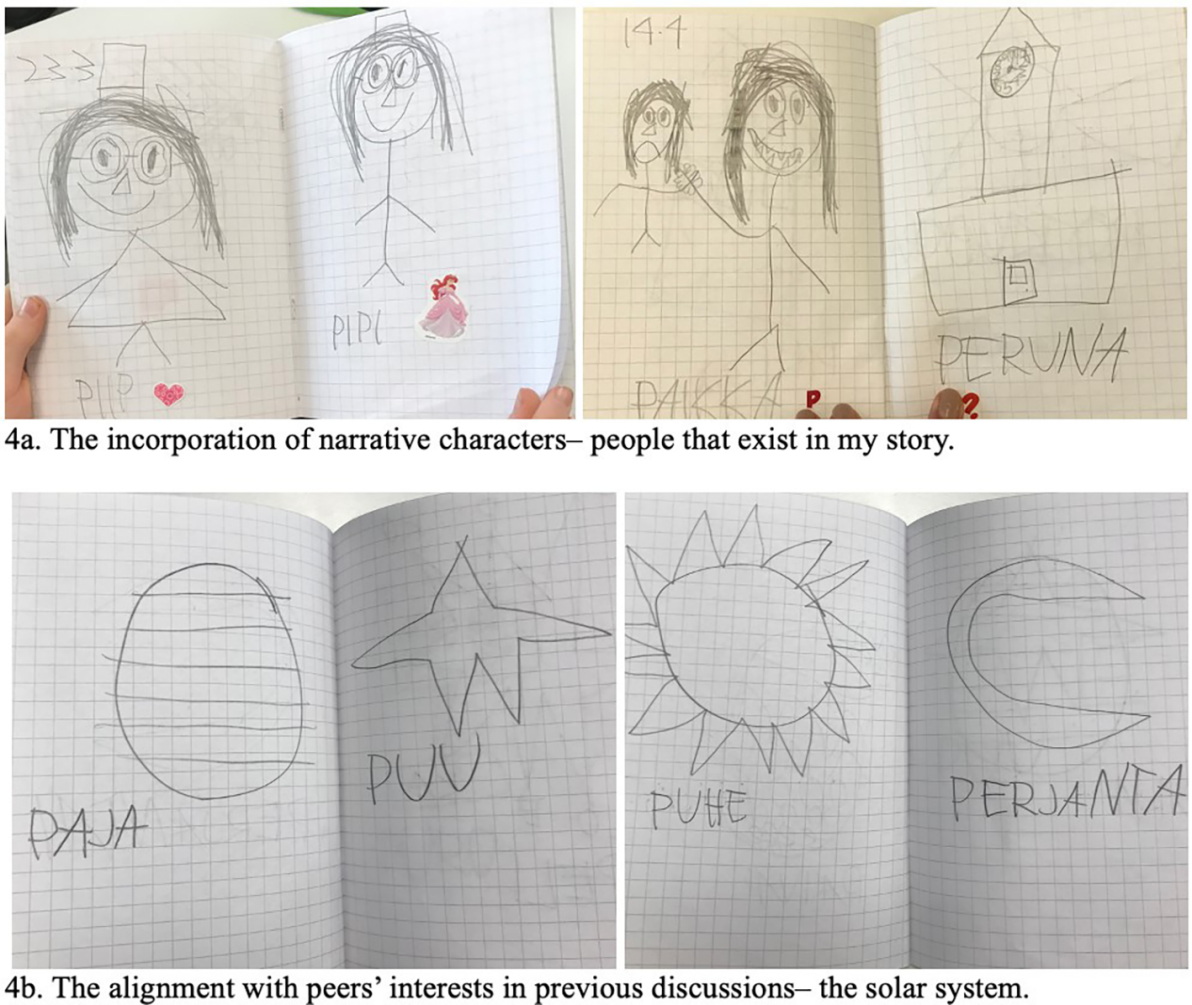
Transformations in Alan’s Diary

Parallel to the repetition, we also observed transformations related to the application of the words and linearity between the themes of Alan’s drawings and the content brought by other children during the morning circle. Looking at the diary longitudinally, we notice that further in practice, the use of words became more intentional, and combinations of related words appeared together. For example, “*pähkinä and pähkinäkana*/ nut and chickennut or *paju and pajunkoskyosi*/willow and willow tree”. The drawings also occupied an entire page, referencing narratives involving people (see Figure 4a), which created more engagement from classmates during Alan’s presentation. Children move towards him in the circle and request more time and a better angle to look at the drawings.

Additionally, we identified similarities between the child’s entries and the themes discussed by peers in morning circles. Alan started incorporating into his diary elements of topics brought by his peers, showing that he had been attentive to group discussions even if not verbally engaged in initiating inquiries and discussion. Alan is participating the construction of group knowledge and the group interest is shaping his (see Figure 4b).

By sharing his diary in morning circles, Alan communicates engagement, creating common grounds, shared interests, and material participation in the group—they are learning about the solar system together. All these transformations within the repetitive patterns in the diary’s construction emerge from the need to keep his identity as a member of the group, and the recordings in the diary support (shape) ways to interact with others in this group process.

**Figure 5:**
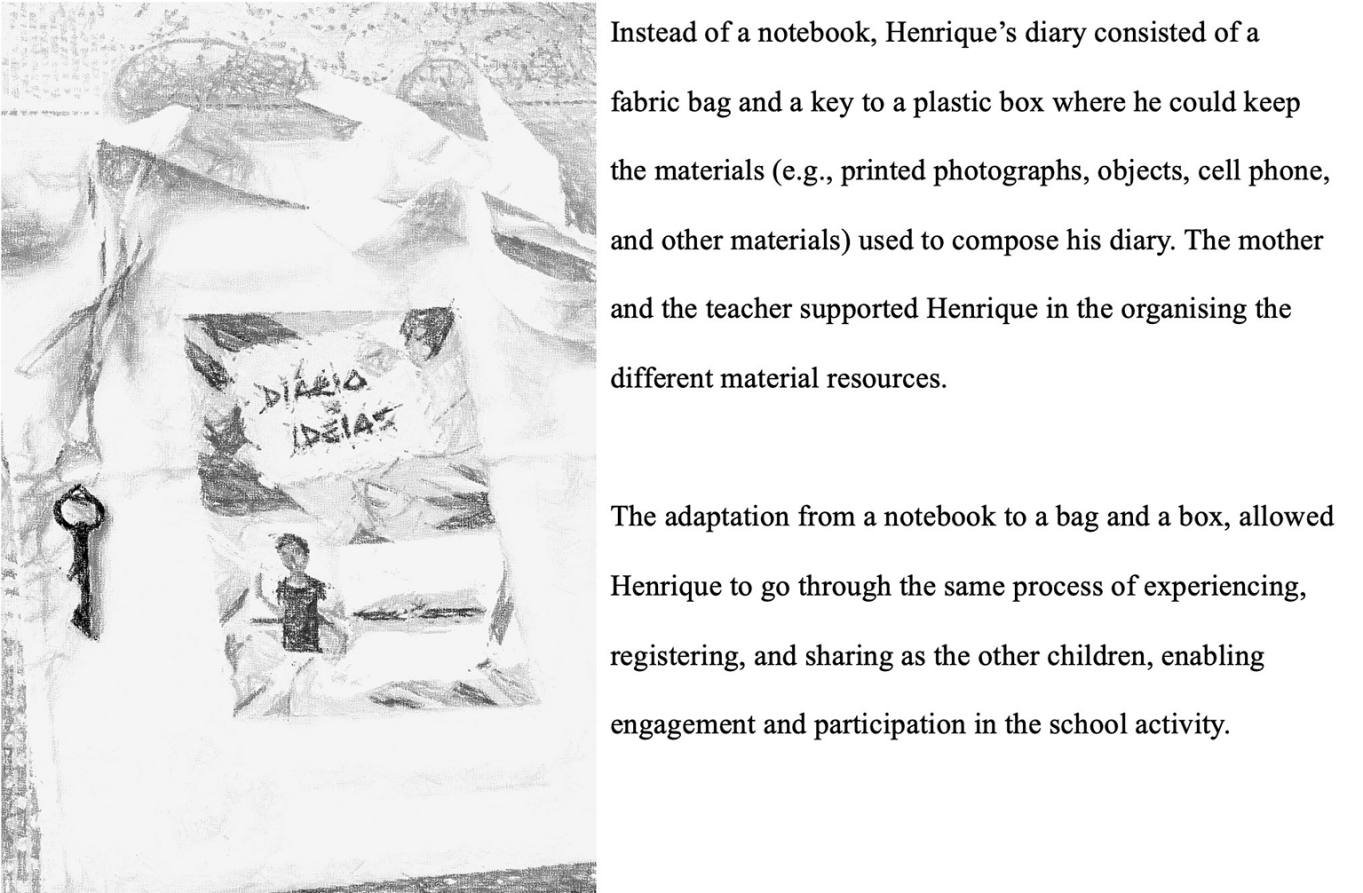
Henrique’s Adapted Diary

In Henrique’s diary, the patterns appear from a different perspective. First, it is important to understand the uniqueness of his diary. During data collection, Henrique did not engage with pencils, papers, or notebooks for the conventional purpose of communicating. Therefore, his diary was constructed with assistance from a family member and contained mainly objects, photographs, and video recordings of diverse moments of his day (see Figure 5). It is important to highlight that the child always initiated the engagement with the diary. Henrique chose the pictures by looking at them on the computer and by expressing different emotional reactions, which indicated to his mother (or to the teacher during school time) the pictures that should be printed. He was also the one who actively glued the pictures into the diary. The same process was applied in choosing videos and music, which then, instead of being included in the diary, was sent to the teacher via Henrique’s cellphone.

**Figure 6:**
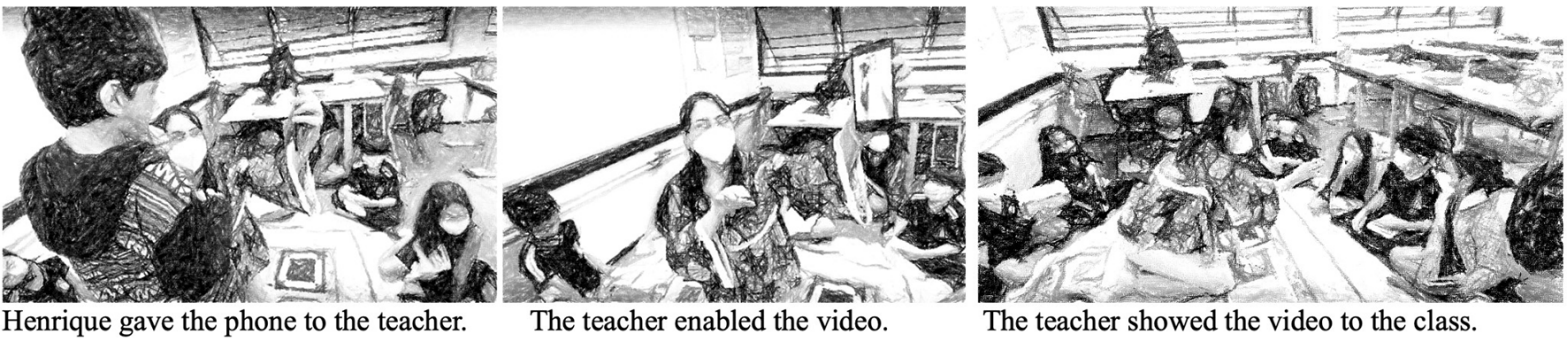
Building Communication Through Image

In the morning circle, Henrique would give the cell phone to one of the teachers to be accessed during the discussion (see Figure 6). The repetition also appeared in the choice of expression for diary entries. Pictures and videos of Henrique listening to music, dancing, and eating were frequent and followed a similar sequence of appearance—first the music, then the dance, then eating-related events (i.e., pictures of him eating or pictures of food itself).

**Figure 7:**
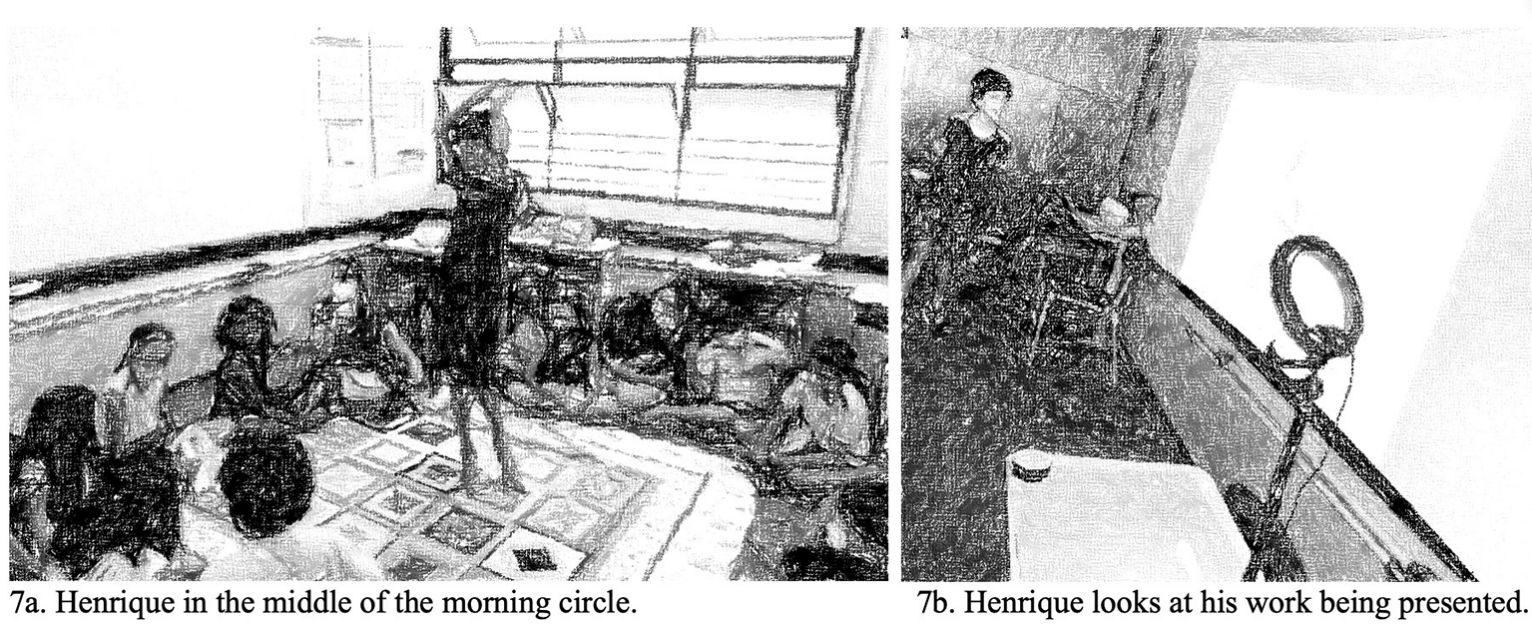
Example of Transformation in the Engagement

We observed, however, that during the use of the diary, through interactions with colleagues, changes occurred in Henrique's behaviours, particularly the gestures and movements he used to signal his wish to participate in the morning circle. At the beginning of the practice with the *Idea Diary*, Henrique mainly walked around the classroom and physically jumped into the middle of the morning circle when he wanted to participate (present his diary or look at someone’s diary) (see Figure 7a) and then left the teacher to present his diary. As the practice continued along the year, Henrique created other behavioural codes for signalling his readiness to share; for example, he clapped the hands of one of the class aids or the teacher (depending on who was holding his diary), creating an engagement that no longer breakdown the flow of the morning circle, but yet defined his time to act. He also changed how he positioned himself during the presentation of his diary. Instead of leaving the scene, he more often positioned himself side-by-side with his pictures or videos or even re-enacted in the scene (see Figure 7b).

Henrique’s case evidences the construction of meaning-making through subtle processes of embodied coordination guided by the willingness, desire, need, or interest in being together.

### Material Engagement Supporting Self-Perception, Shaping Participation, and Enabling Participatory Sense-Making

The engagement with the diary also afforded processes of self-perception and reflection, supporting children to communicate their experiences, ideas, and interests, sharing their subjective productions with the group. In turn, these processes shaped participation and enabled participatory sense-making in small-group interactions.

**Figure 8:**
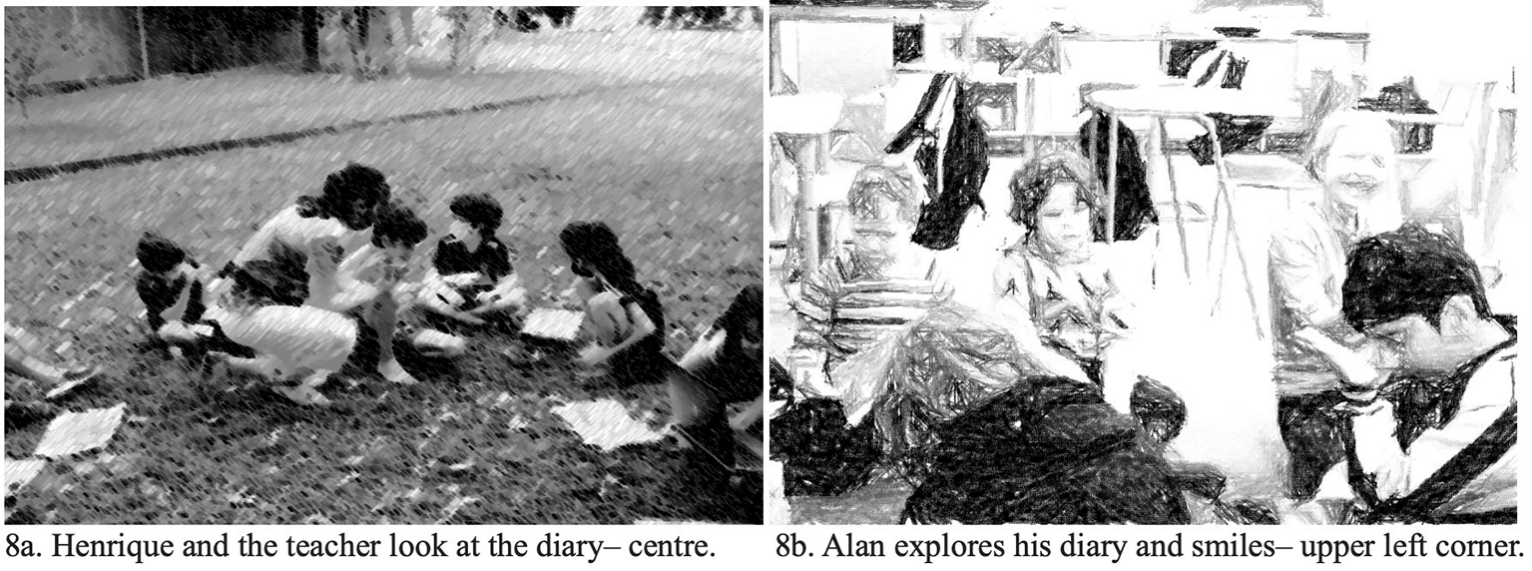
Children Observe Their Diaries

In our analysis, we identified 11 moments when Alan (7) and Henrique (4) spent time looking at their diaries (Henrique supported by the teacher) during the morning circle, and it called to our attention the role of this exploration immediately before they presented their recordings in the morning circle (see Figure 8a–b). Children explored their diaries and re-enacted specific emotional expressions. Alan often laughed and repeated specific words written in the diary; Henrique re-lived his dance movements from the videos or clapped his hands and vocalised expressions when seeing a picture of his family eating. The material engagement with the diary brought to the present moment elements of children’s experiences significant to them, transforming the perceptual and semiotic dimensions of subjective experience through novel multi-modal and recursive dynamics of participatory sense-making. They perceived and recognised themselves in the diary. These moments were, on many occasions, followed by a very clear communication (verbal and non-verbal) of children’s desire to share the diary in the morning circle. Alan and Henrique expressed specific behaviours (e.g., seeking the teacher and clapping her hands—Henrique, and expressing repetitive behaviours—Alan), which influenced the dynamic of the small group discussion, provoking the group to listen to their ideas. We chose two situations to illustrate the implications of this dynamic.

**Figure 9:**
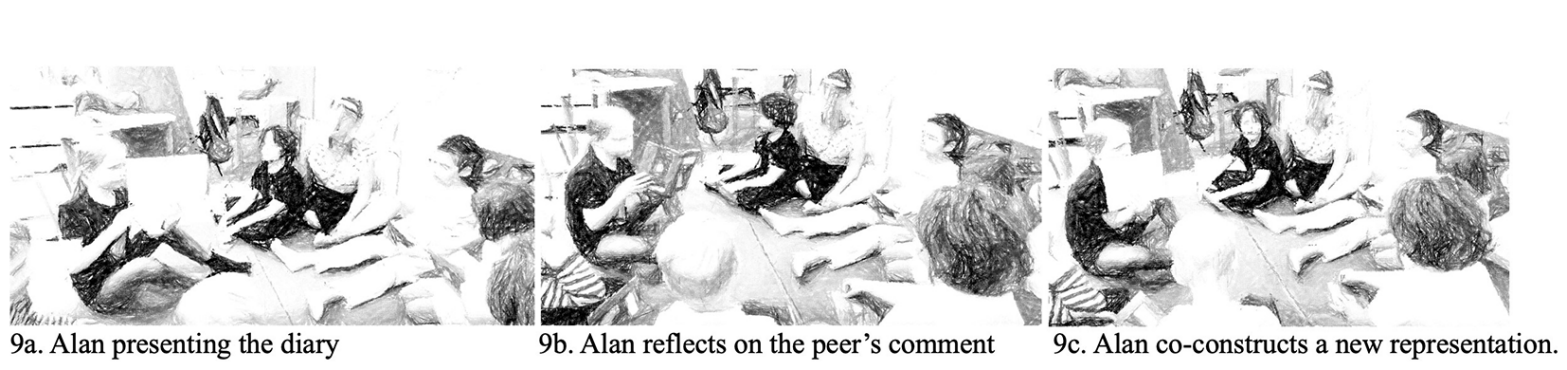
Co-Constructing New Representations

Alan actively participated with new diary entries in all morning circles and always positioned himself sitting together with his peers. Alan sometimes expressed repetitive behaviours (e.g., bouncing back and forth, rolling back on the floor, clapping his hands, and vocalising loudly incomprehensible sounds) that became part of the circle’s dynamic. In one particular meaningful event during the morning circle Number 7 (see Figure 9a–c), Alan held his diary and navigated through the group dynamic, alternating between looking at others’ diaries and looking at his own. The teacher invites Alan to present his diary immediately after he starts expressing repetitive behaviours—bouncing back and forth and clapping his hands. Alan then opens his diary and starts presenting it to his peers: “This is the sun, and this is the moon” (showing the image across the group). A classmate raises his hands and says: “Well, the sun is a little bit more… well, a bit yellow, orange and red”, referring to the inadequacy of Alan’s black and white drawings. Alan looks at his diary quietly, looks back to his classmate, and continues: “And then… here, this is a star. A red star!”, showed the diary to his friends and pointed to another black and white drawing.

In this very brief interaction, we notice that Alan modifies a characteristic of his drawing of a star, co-creating a new perspective of it based on the classmate’s comments about the lack of colour. Alan understood the social dynamic in place and the role of the classmate’s comment and identified a social demand for compromise—for bridging his and others’ ideas. Simultaneously, we also note that the mere indication of the colour in the black and white drawing was enough for classmates to ‘believe’ in what Alan was sharing, and no comments in that regard were made.

There can be several reasons why he includes colour in his drawing to meet the classmate’s needs for other forms of visual expression (colours). It can be to ensure group engagement or to keep aligned with the peer's observation, for example. However, this episode shows that Alan is co-creating, giving new meaning to his ideas—a participatory sense-making particular to this moment of interaction.

Henrique was present in all morning circles. When the practice of the *Idea Diary* started, Henrique established few communication processes with classmates, resorting to repetitive movements of picking up school objects and putting them in their mouths. In most situations, Henrique walked around the classroom during the morning circle, giving attention to the narratives only when specific themes of his interest were mentioned. With time, Henrique approached the circle and kneeled beside his classmates to listen to the stories. When it was time for Henrique’s participation, the teacher called him, and together they went through his materials. For Henrique, seeing himself in the pictures and videos was part of engaging with the morning circle activity. Henrique skimmed through his diary with the support of the teacher or class aids, expressed smiles and pointed with his finger at the photo he would like to show to others. While the teacher showed the photo to Henrique’s classmates, he observed children’s faces.

Analysing this dynamic through microanalysis, we identified meaningful events where Henrique and his peers, together, extend a behavioural reference from Henrique’s diary to the classroom context, co-creating within the interaction a new communication sign for Henrique and the class. For example, in one of the morning circles, when the teacher shared a video of Henrique singing with his mother, children observed that Henrique clapped his mother's hands often, using the behaviour to communicate with her while dancing to the song. When the video finished, one of the classmates stood up and walked towards Henrique, reaching out her hands in a similar gesture as in the video. Henrique smiled and then lightly clapped his hands on his classmate’s and looked at her. He then started to re-enact the movements in the video euphorically (i.e., large movements and loud sounds), and his peer copied his movements.

**Figure 10:**
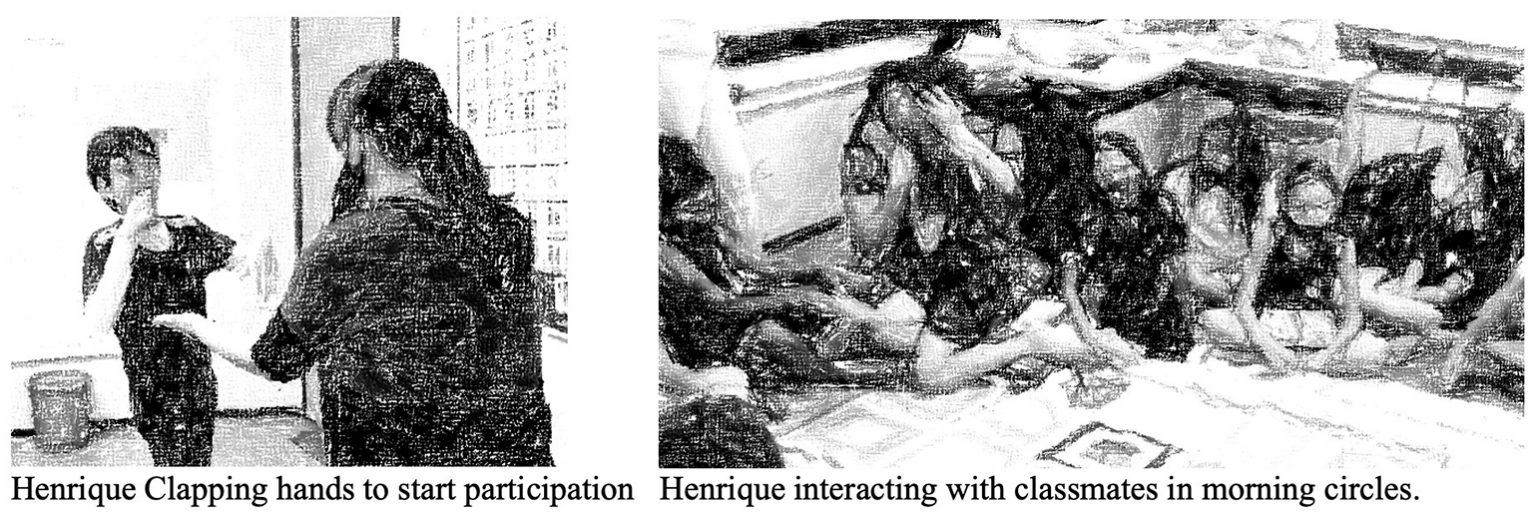
The Creation and Use of a Co-Constructed Embodied Communication

In this brief event, children used an element of Henrique’s emotional expression represented in his diary to create an embodied language of engagement that could be used to facilitate their communication. This vulnerable dynamic emerged in the living moment of the interaction between Henrique and his peer, enabling them to participate in sense-making of the clapping behaviour and set out a communication channel further used by the entire classroom, including the teacher (see Figure 10). Walking towards the teacher to clap on both her hands, establishing a sign of interaction, and demonstrating being present and willing to participate became an embodied communication process in all future morning circles.

In another moment, further on the diary practice, we identified a second meaningful event showing an interesting emotional expression triangulation. The event starts with Henrique soliciting participation in the circle (he walks towards the teacher and claps his hands on hers). The teacher shows the cell phone to Henrique and asks if he wants to choose what they will show to the class. Together, they scan through the pictures. Henrique looks attentively and smiles, indicating the picture he wants to show. Aware of his emotional experience represented by the picture, Henrique anticipates the engagement of his peers, who are now used to seeing his diary through the phone's lenses. Henrique pushes the teacher’s hands towards the group of children and looks at them. The teacher verbally checks the meaning of Henrique’s movements. “*Do you want to show this picture? I will show this photo to the class (shows the picture).*” Children smile, laugh, and comment on the picture, narrating what they see and think about Henrique’s experiences. For example, children stated their perception of what Henrique was doing: “he is dancing very well,” or reflected on his feelings, “*do you think he is happy when he dances?*” and the overall impression of the situation, “*that is so funny*”. When looking at Henrique’s pictures, children's expressions of emotionality engage Henrique, who then expresses similar signs of emotionality as his peers (smiles and laughter). Together, they re-enact the experience in the picture, creating a shared emotionality unique to the lived experience in this small group.

In both cases, the material engagement with the diary enabled children to express themselves and join the group’s dynamic without verbalisation, connecting them with the classroom context and the sharing dynamic supported by the diary practice. Engaging with the diary shaped Alan’s and Henrique's interaction with his classmates, teachers and class aids, creating an entire set of communicative resources.

## Discussion

The present study explored material engagements and their role in supporting the participation of children on the AS in small-group learning situations. By looking at elements of children’s subjectivity expressed through their recordings in the diary and by adopting an enactive view in the analysis of the social interactions in which the diary is presented and discussed, we examined how children on the AS engaged in the activity and, in doing so, materialised participation in small-group discussions. To discuss our findings, we highlight three aspects of this process relevant to understanding further the role of material engagement in the sociality of people on the AS and the practical applications of enactive approaches in educational psychology research and educational practices.

The first aspect relates to comprehending repetition as an element of children’s subjectivity and, within this perspective, *understanding engagement through repetition*. Repetitive behaviours and interests are commonly associated with autism (American Psychiatric Association, 2013; Bottema-Beutel et al., 2015) and can appear both through linguistic echolalic utterances (De Jaegher, 2013) and in the repetition of a specific structure or a particular topic in the interaction (Dobbinson et al., 1998). Previous studies with children on the AS have called attention to possible repetitions of themes in children’s drawings (Ferreira & Bottema-Beutel, 2023), and they have accounted for it as the elements that create regularity, consistency, and familiarity that children on the AS seek in routines. It can be interpreted as fulfilling the same role as the repetitions in their communications (e.g., the palilalia identified in dialogues among people on the AS, Stribling et al., 2007). Our study, however, revealed interesting insights into how repetition in children’s expressions can be recognised as a sign of engagement. For Alan, repetition appeared through the structured sequence of characters and events representing a collection of meaningful elements, words, and interests. He kept the initial idea of the Idea Diary presented through a story to guide his engagement with the diary. For Henrique, it was the recordings of specific experiences which marked how he communicated with others in the world via movements, gestures, and actions. Repetition was not only in using similar materials (black and white drawings on paper) to convey ideas, experiences, or interests, but it is also an element of children’s subjectivity—the way they make sense of the world. Participatory sense-making was possible when classmates engaged with the repetitive characteristics of Alan’s and Henrique’s diaries. This interaction co-created participatory meaning-making unique to this child-material-child engagement. Repetition as an element of the subjectivity of people on the AS and a potential context for participatory sense-making in interactions with people on the AS is not frequently explored.

Still connected to a sense of repetition, our findings suggest that children’s participation became more significant with time. Previous studies have indicated that individuals on the AS derive comfort from the consistency and predictability of situations, particular objects and object types (Forrester-Jones & Broadhurst, 2007). As the experiences with the morning circles repeated and children created a culture around sharing the diary, the predictability of what would happen increased. With it, lesser expectations for unforeseen events in the interactions, which we understand, allowed Alan and Henrique to experience being in the group.

The second aspect we want to discuss relates to the complexity of participation and how material engagement can guide this process, breaking it down. Participation, as previously stated, is a complex phenomenon associated with physical presence and active engagement in a specific situation (Ferreira, 2017). Although several studies have reported how children on the AS can express many complex conversation patterns, indicating the capacity for social reflection (Bottema-Beutel & Kim, 2021; Ferreira & Bottema-Beutel, 2023; Hermans, 2019), most research emphasises the challenges and limitations of this process (Kasari et al., 2011; Ghanouni et al., 2019). The overall idea of participation can often still be associated with a specific situation or environment (e.g., they participate when there is a special pedagogical approach, common interest groups, support services and inclusive environments) (Chan et al., 2023). Nonetheless, by taking an enactive view on participation, this study offers a different perspective on its construction in small groups. Our findings suggest that participatory sense-making happened mainly in situations where children were *aware of and allowed to express themselves*. To better understand the implications of this insight, we call attention to two factors. One is the affordances for action in constructing the diary. Another is the affordances for participatory sense-making in engaging with the diary during morning circle discussions. Engaging in the elaboration of the diary guided a series of actions—perceiving a significant experience, reflecting on it, and recording it in the diary, bearing in mind the sharing process with classmates. It is important to highlight that this series of actions only existed because, or in the context of, the material engagement with the diary. The object and the actions it afforded created the intelligibility of children’s world. It is a complex process of systematising elements of children’s subjectivity—“constitutes a way of understanding reality in which the disordered, contradictory, plural, recursive, singular, indivisible and historical character that characterises this individual can be recognised” (Mitjáns-Martínez, 2005). Within this context of actions, children became more aware of their experiences and had a material platform for expressing the ownership of their ideas and interests.

Simultaneously, engaging with the diary during morning circles afforded the expression of children’s subjectivity. The diary not only stored children's experiences, ideas, and interests but became a prompt for accessing children's affective memories, bridging past and present emotionality. The diary brought forth children’s continuous efforts, making explicit the constellation of subjective senses that inform how they engage and make sense within the world. By engaging with this tool during the morning circle, children re-enacted the experiences and the emotional content connected to them, now in the group context. This complex self-perception, reflection, and communication process is enabled because of the diary and the possibility to manipulate it (look at, scan through, and show to others) during the small-group discussion. It provided the child on the AS the space and voice to externalise their subjective process, to be known by what they can do and who they are and, together with the others, co-construct new experiences and ideas as equals.

Previous research with children on the AS has pointed out the relevance of materials in instructional practices and children’s preferences (Carnahan et al., 2009). However, the focus has been primarily on identifying how children learn better or demonstrate more engagement in the learning process. The present study goes beyond and provides a glimpse into the processes of meaning-making with the group supported through the material engagement with the diary and its structure—*experience, record, and share*, that values children’s subjectivity and allows for gradual transformations in meaning-making processes. The construction of the diary meant something to children; it was led and regulated by their need to self-maintain their identity as a member of that group. Thus providing a new light on the role of material engagement in small group interactions.

Overall, even considering a less radical perspective on material engagement, the insights on how *it shaped participation and enabled participatory sense-making* reveal the communicative competence of children on the AS and how they can be supported in the moment of the interaction. Other empirical studies investigating children’s group learning through the method of the *Idea Diary* have identified the facilitator character of the diary (Guimarães et al. (2022) and have stressed the challenges of school inclusion considering the constraints related to the organisational, political, pedagogical, cultural and subjective nature of the students (Anache & Martins, 2019; Guimarães et al., 2022). To our knowledge, this is the first study that explored material engagement and participation, emphasising the need to integrate multiple subjective aspects of children’s development and learning, especially those with significantly different developmental paths (González Rey, 2011).

### Conclusion

This study joins the current discussion advancing the theoretical embodied frameworks, particularly those guiding a broader understanding of social cognition. It contributes to educational psychology by providing insights into the empirical observations of participatory sense-making in educational practices with children on the AS. At the same time, the findings suggest pedagogical implications related to the potential of the actions developed within the scope of the *Idea Diary* methodology, particularly through the systematised actions of *experiencing, recording, and sharing*. Children become aware of their lived experiences, perceive themselves in the process, recorded their experiences in different social contexts, and then interact to share freely their ideas in morning circles. All of which is done by using different languages, such as videos, drawings, writing and others, enlarging social repertoires. Understanding the role material culture plays in sociality and development is relevant not only in early childhood, where the foundations for many cognitive processes are laid, but in special education, where children with significantly different developmental paths demand alternative ways to communicate, participate, and build knowledge of the world with others.

Although the study targeted only students on the AS, the pedagogical practice used—the Idea Diary, and the methods applied for its analysis can be transported to any educational context. Thus, one of the main contributions of this work for the education audience lies in showing the potential in the analysis of children’s interests, ideas, and productions within the relationship with other students. This perspective offers valuable insights and benefits in multiple ways. It promotes children's well-being by showcasing their abilities, not just their challenges, leading to a more positive self-image. Additionally, it empowers teachers to build on pedagogical approaches centred on the interests of the child on the AS, without the pressure of the standardised approaches that, in different ways, drive comparison.

Overall, we corroborate with the argument raised by De Jaegher (2021) that if we impose a certain understanding of autism and how people on the AS should behave, collaborate, learn, or relate, they cannot in any way be themselves. The challenge placed for teachers, special educators, therapists, and researchers working with people on the AS is to find ways for their subjectivity to be expressed and valued.

### Limitations

Although multiple case studies shed light on the diverse ways a phenomenon can appear, there are limitations concerning the generalisation of our findings. The expressions of children’s subjectivity described in this study are contextualised by the educational practices applied during the study—the Idea Diary and the socio-cultural context of a Brazilian and a Finnish elementary school. The scope of this study was limited to investigating the material engagements from children’s perspectives, excluding the analysis of the teacher’s strategies for conducting the morning circle and overall thoughts on the *Idea Diary* as a pedagogical method. Also, the study reported solely the analysis of a specific set of data—the materials produced by children and the video recordings of the morning circles. The study did not explore the cognitive implications of the material engagement, which could inform further the learning processes. Therefore, future studies should consider a broader analysis of social interactions among these students out of the context of the *Idea Diary*, such as in implementing the classroom activities that can derive from the diary as part of this method. This would provide a more comprehensive understanding of the role of material engagements in learning processes in the school context. Further research could also take a developmental approach and analyse the cognitive role of everyday material engagements with the diary. Lastly, future studies should consider the interposition between children’s and teachers’ viewpoints, as well as a variety of small-group practices that also focus on supporting the participation of children in the AS.
